# Potential for community based surveillance of febrile diseases: Feasibility of self-administered rapid diagnostic tests in Iquitos, Peru and Phnom Penh, Cambodia

**DOI:** 10.1371/journal.pntd.0009307

**Published:** 2021-04-26

**Authors:** Amy C. Morrison, Julia Schwarz, Jennie L. Mckenney, Jhonny Cordova, Jennifer E. Rios, W. Lorena Quiroz, S. Alfonso Vizcarra, Heng Sopheab, Karin M. Bauer, Chhorvann Chhea, Vonthanak Saphonn, Robert D. Hontz, Pamina M. Gorbach, Valerie A. Paz-Soldan

**Affiliations:** 1 Department of Pathology, Microbiology, and Immunology, School of Veterinary Medicine, University of California, Davis, California, United States of America; 2 U.S. Naval Medical Research Unit No. 6 (NAMRU-6), Lima, Peru; 3 Icahn School of Medicine at Mt Sinai, New York, New York, United States of America; 4 University of California Fielding School of Public Health, Los Angeles, California, United States of America; 5 Department of Entomology and Nematology, University of California, Davis, California, United States of America; 6 School of Public Health, National Institute of Public Health, Phnom Penh, Cambodia; 7 Tulane School of Public Health and Tropical Medicine, New Orleans, Lousiana, United States of America; 8 University of Washington, Seattle, Washington, United States of America; 9 University of Health Sciences, Phnom Penh, Cambodia; 10 U.S. Naval Medical Research Unit No. 2 (NAMRU-2), Singapore; George Washington University School of Medicine and Health Sciences, UNITED STATES

## Abstract

Rapid diagnostic tests (RDTs) have the potential to identify infectious diseases quickly, minimize disease transmission, and could complement and improve surveillance and control of infectious and vector-borne diseases during outbreaks. The U.S. Defense Threat Reduction Agency’s Joint Science and Technology Office (DTRA-JSTO) program set out to develop novel point-of-need RDTs for infectious diseases and deploy them for home use with no training. The aim of this formative study was to address two questions: 1) could community members in Iquitos, Peru and Phnom Penh, Cambodia competently use RDTs of different levels of complexity at home with visually based instructions provided, and 2) if an RDT were provided at no cost, would it be used at home if family members displayed febrile symptoms? Test kits with written and video (Peru only) instructions were provided to community members (Peru [n = 202]; Cambodia [n = 50]) or community health workers (Cambodia [n = 45]), and trained observers evaluated the competency level for each of the several steps required to successfully operate one of two multiplex RDTs on themselves or other consenting participant (i.e., family member). In Iquitos, >80% of residents were able to perform 11/12 steps and 7/15 steps for the two- and five-pathogen test, respectively. Competency in Phnom Penh never reached 80% for any of the 12 or 15 steps for either test; the percentage of participants able to perform a step ranged from 26–76% and 23–72%, for the two- and five-pathogen tests, respectively. Commercially available NS1 dengue rapid tests were distributed, at no cost, to households with confirmed exposure to dengue or Zika virus; of 14 febrile cases reported, six used the provided RDT. Our findings support the need for further implementation research on the appropriate level of instructions or training needed for diverse devices in different settings, as well as how to best integrate RDTs into existing local public health and disease surveillance programs at a large scale.

## Author summary

Rapid diagnostic tests (RDTs) are becoming increasingly available and are useful during outbreaks of infectious and vector-borne diseases. This study set out to answer two questions regarding the use of RDTs in community settings. First, how well could community members at two diverse sites–Iquitos, Peru and Phnom Penh, Cambodia–perform an RDT on themselves and willing family members with only written and/or video instructions, and with two tests of different complexity? Second, and only in Iquitos, if RDTs were made widely available at no cost to households, would residents choose to use one for a family member showing febrile symptoms? In Peru, 202 community members were provided written and/or video instructions and a test kit, and then were observed operating the RDT on themselves or family members in their own homes. In Cambodia, 45 Community Health Workers and 50 community members were provided only written instructions at a local health center and asked to carry out the test. In both locations, trained observers scored each of the 12–15 steps necessary to conduct the test on a 3-point scale (1 being lowest proficiency level, 3 being highest), for one of the two RDTs in this study. In Iquitos, >80% of residents were able to perform 11/12 steps and 7/15 steps for the two- and five-pathogen test, respectively. Competency in Phnom Penh never reached 80% for any of the 12 or 15 steps for either test, with the percentage of participants demonstrating high competency ranging from 26–76% and 23–72%, for the two- and five-pathogen tests, respectively. Finally, in Iquitos, RDTs were distributed to households with earlier confirmed cases of dengue or Zika virus: about half of all household members that developed fevers were self-tested or tested by another family member. This study is consistent with our earlier findings from focus group discussions suggesting that implementation of simple home-based rapid diagnostic devices, could be used in different settings, but further research is needed to determine the best type and level of instructions to do this.

## Introduction

As rapid diagnostic tests (RDTs) become easier to use and increasingly available, their application for early detection of disease, especially outside traditional point-of-care settings, has considerable potential for use in disease surveillance programs; for example, options for self-testing during the COVID-19 pandemic. RDTs available for home use have been of particular benefit for blood glucose monitoring in diabetics [1], pregnancy testing [2], and detection of infections associated with social stigmas, such as sexually transmitted infections (HIV, syphilis, hepatitis) [3]. RDTs are also extremely useful for identifying infectious diseases in remote settings without complex equipment, but the potential for their use in community-based or sentinel surveillance programs or incorporation into national treatment programs for diseases other than malaria is largely untapped [4].

Unlike pregnancy tests or in-home HIV tests, which only require dipping a stick in urine or swabbing a gum, RDTs for dengue or other febrile illnesses involve more complex steps such as using a lancet to obtain blood, mixing buffers, and applying appropriate mixtures to the testing device. These latter steps were the primary obstacles for successful use of home-based HIV and malaria RDTs [5–8]. Initially, RDTs for self-monitoring of blood glucose also encountered these problems, but early studies led to test improvements, and these RDTs are now widely used and highly accurate [1].

If reliable and able to be used only with instructions (i.e., no training), home-based RDTs could be used for screening and/or surveillance of both dengue virus, SARS-CoV-2, or other infectious diseases to reduce transmission risk associated with seeking care at health facilities. The impact of home- or community-based RDT use on health systems will depend on validation that untrained community members or lay health workers can use them effectively. The consequences, such as not seeking needed health care for a false negative result or a negative result associated with the timing of the test (i.e., for dengue, too early for the detection of antibodies or too late for the detection of NS1), require consideration prior to widespread use [9]. Similarly, people testing positive must have information on how and where to seek appropriate clinical care for follow up.

In 2012, the U.S. Defense Threat Reduction Agency Joint Science and Technology Office (DTRA-JSTO) initiated a program to develop novel point-of-need diagnostics for surveillance of emerging infectious diseases that included, beyond evaluation of the sensitivity and specificity of the RDTs, a component to assess acceptability and competency for use. Our research carried out under this program was formative and focused on supporting testing of multiplex devices in the DTRA pipeline that included important bio-threats such as plague and melioidosis. Specifically, two RDTs were used in this formative research study: one that detected two pathogens (dengue and *Burkholderia pseudomallei*) and the other that detected five pathogens (dengue, *B*. *pseudomallei*, *Plasmodium vivax* and *falciparum*, *Yersinia pestis)*. In February 2014, focus group discussions with community members and health professionals conducted in Iquitos, Peru and Phnom Penh, Cambodia showed that there was acceptability for at-home use of RDTs in Peru but less enthusiasm in Cambodia, and that a principal motivator for RDT use would be a link to a streamlined process for clinical feedback or follow up based on the test result [9]. There was a strong preference for RDTs to be applied by community health workers (CHWs) in Cambodia, where CHW infrastructure for malaria control was in place in our study communities [9]. Findings from the focus groups were used to guide this next phase of research.

Our objective was to answer the following two questions related to RDT use in dengue endemic settings, represented by Cambodia and Peru. First, could community members (both sites) and CHWs (only Phnom Penh) with appropriate instructions (written and/or video) competently use RDTs and did competency vary with test complexity? Second, would community members (Peru only) use RDTs to self-administer or test a family member if they displayed febrile symptoms? This formative research study was not meant to be generalizable; however, the findings and implications related to this study have broader applications.

## Methods and materials

### Ethics statement

For work in Peru, the study protocol was approved by the U.S. Naval Medical Research Unit No. 6 (Protocol #NAMRU6.2014.0003) Institutional Review Board (IRB), which includes Peruvian representation and complies with U.S. Federal and Peruvian regulations governing the protection of human subjects. IRB Reliance Agreements were established between U.S. Naval Medical Research Unit No. 6 (NAMRU-6), and the University of California, Davis, as well as Tulane University School of Public Health and Tropical Medicine for the NAMRU-6 IRB to be the reviewing IRB. Additionally, the protocol was reviewed and approved by the Loreto Regional Health Department, which oversees health research in Iquitos. For work in Cambodia, the study protocol was approved by the National Ethics Committee in Cambodia and the University of California, Los Angeles IRB. For the first research question involving participant observation while applying the RDT to assess competency with tests of different complexity, written consent was obtained from all adult participants (≥18 years of age) that were either self-applying the RDT, or having another participant administer the RDT on them. Children who participated in the role of “patient” provided written assent (8–17 years of age) and a parent or guardian provided written parental consent for children age 2–17. For the second research question, RDTs were provided to higher-risk households to assess whether people would choose to use them (“RDT adoption” from now on) on a febrile household member. All eligible family members (≥2 years of age) provided written consent to self-administer the RDT or have a family member administer the RDT if they displayed febrile symptoms.

### Study setting

This study took place in Iquitos, Peru, and Phnom Penh, Cambodia, described in detail previously [9,10]. Iquitos is a large urban center (population ~ 400,000) where residents have easy access to a network of government health facilities and hospitals, but they do not have a network of health workers within the city. Although malaria is endemic in villages outside Iquitos, it does not circulate inside the city [11]. At the time of this competency observation study, the Asian-American genotype of dengue virus serotype 2 was circulating and Zika virus had not been detected in the city [12]. Study subjects were recruited between May 2015 and March 2016 from ten city blocks selected at random from four neighborhoods, two with ongoing prospective cohort studies [13–15] and two with no exposure to human research studies to control for the possibility that prior exposure as a research participant might improve competency. Within these randomly selected blocks, our research team recruited participants door-to-door, with one adult per household, until we reached a target of 50 participants per neighborhood. When a participant agreed to participate, our staff would call an administrator who provided the assignment of the two- or five- line device from a randomized list. Related to the question of RDT use adoption, Iquitos has ongoing cohort studies for dengue virus that monitor residents from houses in study neighborhoods three times per week for febrile illness, with subsequent laboratory confirmation if that illness is due to dengue or Zika virus [16,17]. The latter triggers contact cluster investigations where laboratory confirmed cases are interviewed to identify residential locations where the individual spent time in the 15-days prior to infection [14,18,19]. All residents in those locations are then monitored for secondary cases. We leveraged our ongoing cluster investigations to assess RDT use adoption (research question #2) in Iquitos between September 2016-September 2018.

The Cambodian sites were peri-urban areas on the outskirts of Phnom Penh with an active community health worker (CHW) program. CHWs were integrated in both dengue and malaria surveillance programs and provided basic health care to neighborhood residents. During our previous study examining acceptability of RDT use at home, participants revealed a preference for having CHWs apply RDTs in their communities [9]. As a result, both community members and CHWs were selected by convenience sampling, then invited to a local health center to apply the RDTs on others, and their competency was assessed by our Cambodian team.

### Rapid diagnostic devices and application

#### RDT use competency

The two prototype devices used in the observation study were under evaluation by DTRA [20], whose long-term goals were to develop multiplex devices to detect a wide array of pathogens including Biological Safety Level III/IV agents that could be applied directly by community members. Neither device was developed further, but the DTRA-funded observation studies were for these devices, which provided us a model system to test devices of varied complexity. The first RDT identified dengue virus and *Burkholderia pseudomallei*, henceforth called the two-line test; and the second identified five pathogens–dengue virus, *Plasmodium vivax/falciparum*, *Yersinia pestis*, *B*. *pseudomallei—*henceforth called the five-line test. Both devices required collection of whole blood via a finger-stick. The two-line test required 12 steps to complete, whereas the five-line test had 15 steps. As both prototype devices were still being evaluated, and not approved for diagnostic use, the research assistants explained to the participants that there were no meaningful results from these RDTs.

#### RDT Use adoption

For this objective, we used an RDT that was commercially available, the SD Bioline DENGUE Duo (“NS1”), which also performed up to label expectations in a multi-country comparison study [21]. We selected this device because it was approved for use in Peru, providing a diagnostic result to the user–the principal motivator for use. We developed and piloted pictorial and written instructions, an instructional video, and a result reporting form distributed with the device (S1 and S2 Texts).

### Instructional materials

In both locations, detailed systematic instructions with pictures or drawings were developed for low literacy users (S1 Text), piloted in the community prior to use during FGDs, and finalized in both sites–one in real time during the first phase (Iquitos) and one right after the first phase (Phnom Penh)—prior to their application in the second phase. Based on overly complicated instructions that came with the devices, the Peruvian field team developed a one-page document consisting of images and minimal text descriptions of each step. These initial instruction drafts were piloted among the local research assistants, staff, and community members, followed by feedback from FGD participants, who were able try using RDT [9]. The lead scientists from the Peruvian research team (VPS, ACM) led the FGDs, and based on participant feedback and their previous experience producing video instructions [10,22], the potential value of video instructions was recognized; a video was immediately developed and validated in the subsequent FGDs. A DVD containing the final version of the video was included with the test kits to observation study participants (S2 Text. https://youtu.be/5RQtc9KNA5A, https://youtu.be/adv-yQaI_iA, https://youtu.be/arOSOaeulIw). In Cambodia, draft instructions and graphic images were developed from the manufacturer’s instructions, translated to Khmer, piloted with local community members and health center staff prior to their use in FGDs. In contrast to Peru, RDT use by FGD participants was not approved by local IRBs, so adaptation materials in real-time and video development was not possible; instead FGDs used the original piloted written materials.

### Study procedures

#### Objective 1: Could community members in Iquitos and Phnom Penh competently use RDTs of different complexity at home without training?

The rationale for this study was to assess competency of community members or CHWs to apply RDTs without training beyond the written or video instructions provided with the device. After providing consent, participants were given a packet containing one of the devices, all other materials needed to complete testing (including retractable lancets and basic biosafety materials), and instructional materials (written one-page document, and in Iquitos, a DVD with video instructions). Participants were asked to follow the instructions in the packet, while a research assistant, using an observation form (S3 Text), evaluated and scored, on a 3-point scale (1 lowest proficiency, 3 highest), each step in the participants’ use of the device (i.e., lay out materials before starting, determine which finger to prick, use lancet correctly). The observers (research assistants) piloted the observation form among themselves, co-workers (nurse technicians and health center staff), and family members, followed by training sessions where all observers watched the same individual carry out the test, compared and discussed scoring with the lead investigator (VPS), until consistent scoring was achieved among the observers. The research assistants did not interfere or assist the participant unless it was obvious to the observer that an imminent mistake affecting all subsequent steps was about to take place, or if a participant requested help. All assistance was documented.

In Phnom Penh, where FGDs indicated a preference for CHWs applying RDTs over community members, a small trial to evaluate the value of experience or practice for competency was carried out by observing eight CHWs applying the RDT up to six times to determine how long it would take to obtain at least 80% competency. The observers did not interfere or assist the CHWs unless it was obvious that a mistake affecting subsequent steps was about to happen, or if help was requested. That is, real-time feedback was provided between repeat tests only as needed.

### Objective 2: Would community members use RDTs to test their own or family member’s febrile illness? (Peru only)

Participants responding to a hypothetical question about the option to use an RDT for febrile illness are likely to respond with what they perceive to be socially desirable. Hence, to determine whether community members would use an RDT in case of febrile illness, we decided to distribute commercially available dengue RDTs to families perceived to be at high risk for dengue or Zika based on proximity to other confirmed dengue or Zika cases in their communities. Leveraging the research infrastructure in Iquitos, where our research team has been conducting contact cluster investigations since 2008 [14], we identified laboratory-confirmed dengue or Zika cases (“index cases”), then carried out an interview to find out what locations the infected person had visited during the 15 days prior to becoming ill [23]. We then visited the index case’s home and contact locations, and provided a kit with two RDTs (SD Bioline Dengue Duo), written and video instructions (S1 and S2 Texts), and all additional materials needed to carry out the test to each consenting household in the cluster. The kits were removed from each house after a 30-day follow up period if not used. During the follow up period, each household was visited every 1–2 days to identify new febrile cases which were tested by PCR. Our team carried out confirmatory testing if the person used the RDT. We recorded how many individuals showed symptoms of fever, whether they used the RDT, and if not, explored their rationale for not using the RDT.

### Analysis

Research assistants provided a score, ranging from one (unable to complete the procedure without asking for help) to three (able to complete the step correctly without assistance) for *each individual step* needed to complete the test (12 steps were scored). An intermediate score of two was given when the participant demonstrated some degree of competency but needed some assistance or reassurance. To assess factors contributing to correct use of the RDT, we developed an overall “competency score” for each participant’s use of one of the two RDTs. The scores for each of the 12 steps that both tests had in common were summed for an overall competency score (range 12–36) and divided by the 12 common steps evaluated (range 1–3). A binary response variable of > 2.9 defined as “highly competent” was used to construct separate logistic regression models for Peru and Cambodia. All analyses were conducted in SAS/STAT software, version 9.4 of the SAS system for Windows (SAS Institute, Cary, NC).

## Results

### Participant characteristics

The demographic characteristics of the study participants who applied an RDT are summarized in Table 1. In Peru, only community members were enrolled in the study; 203 participants were originally observed using an RDT, and one observation was excluded because the participant was a NAMRU-6 nurse technician with extensive experience using RDTs. In Cambodia, due to a stated preference for relying on CHWs, we observed RDTs being applied by both CHWs (n = 45) and community members (n = 50). In Iquitos, females comprised a significantly higher percentage of the participants and those recruited were significantly younger than in Phnom Penh (gender: χ2 = 4.5, df = 1; p = 0.0349; age: χ2 = 26.5, df = 1 p<0.0001). Education levels, however, were higher overall in Peru than Cambodia (χ2 = 96.2, df = 2; p<0.0001). Forty-one percent of the participants were housewives in Peru compared to 17% in Cambodia (χ2 = 16.5, df = 1; p<0.0001), where most of the participants were low-skilled laborers. The majority of RDTs were self-administered in both countries, but when administered on others, they were done on family members and friends, including young children (as low as 5 years of age).

**Table 1:** Summary of study population of device application by community members (excluding the 8 Community Health Workers in Cambodia that participated in a separate experiment described below).

Characteristic	Options	Peru	Cambodia
Participant	Community Health Worker	--	45 (47.4%)
	Household Resident	202 (100%)	50 (52.6%)
RDT	Two Line Test (12-steps)	101 (50.0%)	42 (44.2%)
	Five Line Test (15-steps)	101 (50.0%)	53 (55.8%)
Gender*	Females	142 (70.3%)	55 (57.9%)
	Males	60 (29.7%)	40 (42.1%)
Age (years)**	18–29	84 (41.6%)	24 (25.3%)
	30–39	49 (24.3%)	11 (11.6%)
	40–49	32 (15.8%)	19 (20.0%)
	50–59	21 (10.4%)	27 (28.4%)
	>60	16 (7.9%)	14 (14.7%)
Education**	None	(0.0%)	3 (3.2%)
	Primary	7 (3.5%)	41 (43.2%)
	Secondary	94 (46.5%)	41 (43.2%)
	Technical	55 (27.2%)	(0.0%)
	University	46 (22.8%)	10 (10.5%)
Occupation**	Housewife	82 (40.6%)	16 (16.8%)
	Student	22 (10.9%)	9 (9.5%)
	Sales	20 (9.9%)	18 (19.0%)
	Health/Education	21 (10.4%)	2 (2.1%)
	Skilled Work	7 (3.5%)	10 (10.5%)
	Police/Military	1 (0.5%)	3 (3.2%)
	Unqualified	14 (6.9%)	8 (8.4%)
	Driver	1 (0.5%)	4 (4.2%)
	Office work	7 (3.4%)	12 (12.6%)
	Other	27 (13.4%)	13 (13.7%)
RDT Tested on	Self-Administered	115 (56.9%)	50 (52.6%)
	Adult Family Member	50 (24.8%)	33 (34.7%)
	Child Family Member	29 (14.4%)	1 (1.1%)
	Friend/Other	8 (4.0%)	11 (11.6%)

*p-value = 0.0349

**p-value<0.0001; for age the 18–29 y/o category was compared to ≥30 years of age; for education categories were collapsed to none/primary, secondary, and university/technical; for occupation categories were housewives versus other professions.

In Iquitos, >80% (range 80–95%) of residents testing the two-line test were able to perform 11 out of 12 necessary steps competently without requesting help (scored a ‘3’ [high in Table 2] by a trained observer). The most challenging step was using the retractable lancet (69% used the lancet correctly without help) (see Table 2). The steps associated with starting the procedure (recognizing test materials) and blood collection (locate puncture site, capillary tube use, blood collection) required more help than the remaining steps. The five-line test, with additional steps, was more difficult for residents than the two-line test. High competency was observed for >80% (range 80–91%) for only 7 of 15 steps evaluated.

**Table 2:** Observations of community members’ competency applying two rapid diagnostic tests (two-line, five-line) in Iquitos, Peru and Phnom Penh, Cambodia. The number of participants scored in each competency category, high = 3, medium (med) = 2, and poor = 1, are shown for each testing step.

Testing step (Two-line test)	Level of competence observed when applying RDT							
	Peru (n = 101)				Cambodia (n = 42)			
	Poor	Med	High	%High	Poor	Med	High	% High
Recognizes materials	0	20	81	80.2	4	27	11	26.2
Locates puncture site	0	16	85	84.2	5	7	30	71.4
Cleans site	0	6	95	94.1	5	8	29	69.1
Lancet use	1	30	70	69.3	11	4	17	40.5
Capillary tube use	0	18	83	82.2	11	12	19	45.2
Blood collection	1	15	85	84.2	9	11	22	52.4
Locates well	0	8	93	92.1	4	8	30	71.4
Blood transfer	0	9	92	91.1	5	5	32	76.2
Adds buffer	0	5	96	95.0	7	13	22	52.4
Waits for result	2	4	95	94.1	4	10	28	66.7
Reads result	0	9	92	91.1	8	7	27	64.3
Marks result on paper	1	6	94	93.1	8	9	25	59.5
**Testing step (Five-line test)**	**Peru (n = 101)**				**Cambodia (n = 53)**			
	**Poor**	**Med**	**High**	**% High**	**Poor**	**Med**	**High**	**% High**
Recognizes materials	0	37	64	63.3	5	36	12	22.6
Add reagent to tube	0	28	73	72.3	--	--	--	--
Locates puncture site	1	28	72	71.3	6	17	30	56.6
Cleans site	1	18	82	81.2	2	17	34	64.2
Lancet use	0	36	65	64.4	14	20	19	35.9
Capillary tube use	1	42	58	57.4	20	21	12	22.6
Blood collection	5	47	49	48.5	8	26	19	35.8
Blood transfer to tube	1	21	79	78.2	--	--	--	--
Mix blood in tube	1	31	69	68.3	--	--	--	--
Locates well	0	20	81	80.2	11	14	28	52.8
Blood transfer	0	19	82	81.2	8	22	23	43.4
Adds buffer	0	12	89	88.1	9	16	28	52.8
Waits for result	0	10	91	90.1	5	10	38	71.7
Reads result	0	12	89	88.1	7	9	37	69.8
Marks result on paper	2	14	85	84.2	10	14	29	54.7

In Phnom Penh, users universally struggled to perform the required tasks (every category, both devices). The percentage of participants demonstrating high competency on their ability to execute the steps appropriately without asking for help, ranged from 26–76% and 23–72%, for the two-line and five-line tests, respectively. As in Iquitos, Phnom Penh participants had more problems with the initial steps of the procedures, specifically, recognition of the test kit materials and blood collection steps (Table 2). Although CHWs tended to do slightly better than community members, the overall difference was not statistically significant, so the aggregated results are presented in Table 2.

To assess factors contributing to correct RDT use, we developed an overall “competency” score for each participant’s use of one of the two RDTs. For each of the steps observed, the scores were summed and divided by the number of steps assessed, providing a final competency score between one and three. The competency scores were higher overall in Peru, ranging from 2.00 to 3.00 (IQR 2.75–3.00), than Cambodia where scores ranged from 1.08 to 3.00 (IQR 1.92–2.75). To examine factors associated with competency, we created a dichotomous variable for competency: high (competency score >2.9) and low to medium competency score ≤ 2.9).

In, Iquitos household members performed the two-line test significantly better than the five-line test; the odds of a high competency score were 4.00 (95% CI 2.13–7.54) for the two-line test compared to the five-line test. Younger household residents (18–34 years of age) were 2.28 (95% CI 1.21–4.28) times more likely to perform the test at a high level than older household residents (34–78 years of age). Education level was also an important factor; people reporting any post-secondary education (university or technical school) were 2.69 times (95% CI 1.45–5.00) more likely to perform the test at a higher competency level than participants reporting primary or secondary education only.

In Cambodia, no significant difference in ability to perform the test was observed between the two devices, between CHWs and householders, or by age. Only education level had a statistically significant effect: participants with post-secondary education were 12.49 times (95% CI 1.17–133) more likely to perform the test at a high competency level compared to participants with less education (none, primary, or secondary).

To assess how many repetitions of the test would be necessary to perform the test competently, eight CHWs (different from those who participated in the earlier study) carried out the two-line (n = 4) and five-line (n = 4) tests up to six times on different individuals. The only feedback provided was during the testing process, when the observer intervened if requested or a significant mistake was imminent. As expected, the learning curve was fast; by the sixth trial, all four CHWs using the two-line RDT had perfect scores in all steps, without needing any help. The steps that required the most practice were waiting for results (one took five attempts before doing this correctly), reading results, capillary tube use, and blood collection (three CHWs took four attempts before doing these steps correctly). Scores also dramatically improved for the five-line device, although one CHW still struggled with reading and interpreting the results even at the sixth attempt. Additional steps that required various attempts prior to achieving perfect scores were blood collection, blood transfer, adding buffer, and waiting for results.

### Use of commercially available RDTs by community members with febrile illness (Peru only)

In Iquitos, we provided FDA approved and commercially available SD Bioline Dengue Duo test kits (NS1 test), including all the items needed for their use and written and video instructions, to 74 households participating in 56 cluster investigations [14]. Seventy-four (72%) of the 103 houses consented to participate. Of these 74 households, 17 of the clusters were initiated by a dengue case, and 39 clusters were initiated by a febrile case that was negative for dengue but with a presumptive or laboratory confirmed Zika case. When Zika virus was circulating (June 2016 to April 2017), we explained to the participants that there was still a possibility that any febrile illness could be dengue, but that the test might also be positive for Zika because of cross reactivity [24]. Within participating households, 293 of 445 (66%) residents provided consent. Out of these 74 households and 293 individuals consented, there were 14 reports of individuals who developed dengue-like symptoms, from 10 households, that could have used the NS1 test. Of these, seven of the NS1 tests were applied to six individuals: one on an adult, five on children between the ages of three and 13, and in one participant, a second test was repeated by the family member. Eight individuals who could have used the available test in their home decided not to use it.

The primary reasons for not using the NS1 test were fear of performing the test incorrectly or “wasting it”, or they reported a lack of fever with their other symptoms so they assumed it was Zika and should not use the test. They preferred to wait for the phlebotomist who was part of our research team to test them rather than self-administer the test. One individual forgot that they had the test altogether.

## Discussion

Our study provides evidence that home-based RDT use (with RDTs of limited complexity) may be feasible in certain communities, but less so in others, depending on the demographic and educational characteristics of the populations that would use them. Additional research on the necessity of training and locally adapted RDT implementation strategies are needed before their incorporation into public health programs. For example, in our Cambodian site, neither community members or CHWs were able to apply the RDT tests with sufficient competency to justify their deployment in this site without training, but repeated use among CHWs improved performance suggesting that with training, use by CHWs (and possibly community members) could be feasible.

In locations where CHW programs are in place, the advantages of providing formal training to volunteers who are already trusted by their community and have established connections with the health care system could outweigh any of the perceived benefits from home based RDT use–and this is what is done successfully for malaria RDT programs, for example [25]. CHWs could easily receive dedicated RDT training with refreshers, carry and apply RDTs regularly, interpret tests correctly, and ensure that a patient receives proper follow-up depending on the test results. Overall, there is considerable evidence that CHWs improve health outcomes and reduce health disparities, and when appropriate, should be promoted in communities around the world, particularly in areas where infectious diseases are endemic [26].

CHW programs, however, are not universally available, as in Iquitos, and there are circumstances where home-based testing may be more appropriate or justified. For example, for diseases with a stigma (i.e., HIV and other sexually transmitted diseases) or associated with high mortality or are highly contagious (i.e., COVID-19 and Ebola), home-based RDT strategies without training may be necessary and the most appropriate option to address privacy and/or safety concerns. Our study directly tested the feasibility of home-based testing of two prototype devices without the benefit of training.

Competency was higher for the less complex two-line test compared to the five-line test in both countries, but this difference was only statistically significant in the Peru site, where performance of community members was sufficiently high to consider future deployments. Overall, “lancet use,” “capillary use,” and “blood collection” were the categories in which participants had the most difficulties. For example, for the two-line test, only 69% and 43% of the participants were able to use a retractable lancet correctly in Iquitos and Phnom Penh, respectively. The challenge of extracting blood by lancet has previously been reported for in-home HIV RDTs [27]; considerations about types of lancets and clear instructions are necessary to make it more user-friendly. The five-line test, performance was worse because of the additional steps of collecting the blood from the finger, mixing it with a buffer, and then using a different pipette to transfer this blood and buffer to the RDT. Home-based RDTs should be designed to have as few simple steps as possible, and specific devices must be tested before wider deployment. Interest in multiplex tests is attractive but needs to be balanced with simplicity for deployment to remote sites.

This study found that the enthusiasm expressed by study participants from Iquitos for using “dengue tests” was consistent with that observed in prior focus group discussions carried out in both sites [9]. During the current study, some of our Peruvian participants expressed a sense of empowerment using the RDTs. Despite their initial fears, they were pleasantly surprised and proud of their performance. Our study nurse noted that small children were willingly let their mother stick their finger even though they would cry during the procedure at hospital or health facility. Also, in Iquitos, competency did not appear to be influenced by having prior exposure to participating in a research project, as we recruited study participants evenly from neighborhoods with and without ongoing research studies.

Instructional and training strategies are key issues to examine in the context of future implementation. The first step would be to conduct a rigorous site-specific validation of instructional materials. Circumstances in each study site resulted in different instructional materials for RDT users–including the availability of a video in Iquitos. The role of the video instructions in the observed differences in competency between Iquitos and Phnom Penh warrant further investigation: did the video alone make a big difference between sites? With the explosion of “YouTube” instructional videos, and in the context of possible infectious disease for which isolation is important, the value of providing this type of medium for instructions is possible. Future studies should include an evaluation of different types and levels of instructional materials with and without the inclusion of formal training.

Also critical is the identification of what would motivate home or community based RDT use in diverse settings. In Peru, it was the possibility of streamlining services once the patient reached health facilities [9]. An RDT dengue result would be useful to a physician but clinical follow up should be sought independent of the test result. Our participants were hopeful that a preliminary test would reduce the wait time and number of steps once seen at a health facility [9]. There are many potential programmatic benefits of home-based RDT testing for dengue virus infection if this testing could be incorporated into government surveillance or outbreak response. One relevant application could be for locations where COVID-19 and dengue are co-circulating and cause a case management conundrum. For example, in Iquitos, prior to the COVID-19 pandemic, suspected dengue cases were encouraged to present to health care facilities for clinical monitoring, a practice that should now be discouraged unless patients have dengue warning signs [28]. In these current circumstances, a resident with a dengue positive test result could potentially be directed to a dengue specific facility. Coordinating telemedicine strategies with home RDTs could potentially be used for triage of dengue and COVID-19 patients, reducing contact at health facilities. Policies surrounding home RDTs are yet to be discussed on the large-scale but could provide innovative strategies for navigating the COVID-19 emergency, or others in the future.

A principal limitation of this study was the ability compare RDT competency between Peru and Cambodia, because the two sites had different instructional methods (written/graphical information versus video), implementation strategies (community health workers versus community members), and contexts (culturally, geographically, and even types of diseases and health systems). Training of the personnel carrying out the observations was done by country specific teams with the same reporting format, but we recognize that some scoring differences were possible. For this reason, we have avoided direct comparisons between the two countries when possible since this was not the focus of the study.

Another important study limitation was determining if Iquitos residents would use an RDT if they were provided at no cost. Dengue transmission rates were low during the study period, resulting in an inadequate sample size to draw meaningful conclusions; there were only 14 febrile participants with access to home RDT testing, of which six used the RDT. Our contact cluster study design maximized the possibility of finding febrile contacts, but these households had constant contact with study personnel which may have biased residents’ decision to use them. Regular visits from our staff reminded people to use the tests, but was also one of the most frequently cited reasons not to use the tests (why use the test when someone trained would do it the next day?) Also, complicating this study was the requirement to have informed consent ahead of time. All these factors made it difficult to test home-based RDT use under real-life circumstances.

In conclusion, we found that in Peru, using a simple device, a majority of community members using instructions, but no training, were able to competently use an RDT on themselves or someone else. RDT competency, however, was not achieved for the device with more complex steps or for either device in Cambodia. Further research is needed to determine the level and type of instructions needed that is locally appropriate contrasted with training of key community members (volunteers, pharmacy workers, school nurses, or health promoters), and how to best integrate RDTs into different health systems before use or scale up. The potential applications of home-based RDT use are numerous, in particular, assisting in managing outbreaks, especially when there is a need to reduce contact with health facilities to a minimum as observed during the COVID-19 pandemic.

## Supporting information

S1 Text Written Instructions provided to community or health workers (Cambodia only) to perform self-testing. (PDF)

S2 Text Video instructions provided to community members on DVD to perform self-testing for two-line (https://youtu.be/5RQtc9KNA5A), five-line (https://youtu.be/adv-yQaI_iA) used for observational competency studies and the SDBioline Dengue Duo https://youtu.be/arOSOaeulIw. (DOCX)

S3 Text Observational data collection sheet used by study observers. (DOCX)

S1 Raw data Raw data set (Raw_data.xlsx) used for Tables 1 and 2 and competency score analyses. (XLSX)
