# Supplementary material for: Potential for community based surveillance of febrile diseases: Feasibility of self-administered rapid diagnostic tests in Iquitos, Peru and Phnom Penh, Cambodia
Source: PLoS Negl Trop Dis. 2021 Apr 26;15(4):e0009307. doi: 10.1371/journal.pntd.0009307 (PMC8101991; doi:10.1371/journal.pntd.0009307)
Supplement: S1 Text — (PDF) [file pntd.0009307.s001.pdf]

S1. Written Instructions provided to community or health workers (Cambodia only) to perform self-testing.

CONTENTS

1. **Written Instructions for two-line test (Cambodia)**
2. **Written Instructions for two-line test (Peru)**
3. **Written Instructions for five-line test (Peru)**
4. **Written Instructions for SDBioline Dengue Duo (Peru)**

របៀបធ្វើតេស្តឈាមរហ័ស រកមេរោគគ្រុនឈាម-មេលីយ៉ូដូសីស

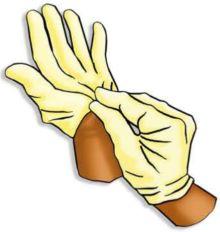

១-ពាក់ស្រោមដៃ។

២-ជ្រើសរើសកន្លែងជោះឈាម។ ឧទាហរណ៍នៅចុងម្រាមដៃ។

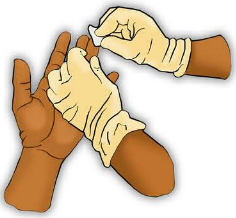

៣-សំអាតស្បែកនៅកន្លែងដែលបានជ្រើសរើសសំរាប់ជោះឈាម ដោយប្រើសំឡីអាល់កុលដែលមាននៅក្នុងកញ្ចប់។ទុកអោយស្ងួតមុននឹងជោះឈាម។

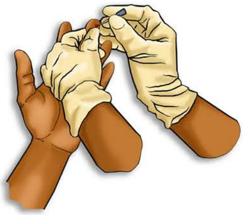

៤-បើកគំរបម្ជុលសំរាប់ជោះឈាម។

៥-សង្កត់ម្ជុលអោយខ្លាំងនៅលើកន្លែងដែលបានជ្រើសរើសសំរាប់ជោះឈាម។

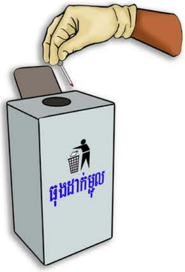

៦-បោះម្ជុលជោះឈាមចោលក្នុងធុងសំរាមសុវត្ថិភាពសំរាប់ដាក់ម្ជុល។

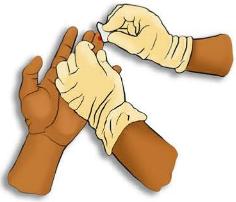

៧-ជូតឈាមដែលមើលឃើញជាមួយនឹងដុំសំឡីស្អាតគ្មានមេរោគ។

៨-យកបំពង់បឺតឈាមមួយ។

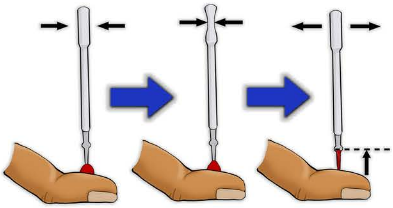

៩-ច្របាច់ចុងបំពង់បឺតឈាម ហើយដាក់វានៅលើកន្លែងដែលបានជោះហើយ។ឈាមនឹងហូរចូលក្នុងបំពង់។បន្តបូមឈាមពីកន្លែងដែលបានជោះ រហូតទាល់តែឈាមហូរពេញបំពង់។អ្នកអាចច្របាច់បន្ថែមដើម្បីអោយឈាមហូរចូលពេញបំពង់។

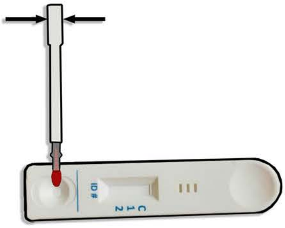

១០-យកបន្ទះតេស្តចេញពីកញ្ចប់ ហើយដាក់លើកន្លែងរាបស្មើ។

១១-បង្ហូរឈាមទាំងអស់ចេញពីបំពង់បឺតឈាមដាក់ចូលទៅក្នុងរន្ធសំរាប់បន្តក់ឈាម ដោយយកចុងបំពង់ប៉ះថ្នមៗជាមួយរន្ធសំរាប់បន្តក់ឈាម។

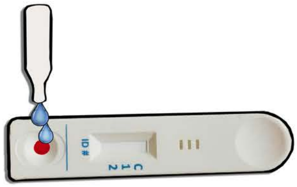

១២-បន្ថែមទឹកថ្នាំ ២តំណក់ចូលទៅក្នុងរន្ធសំរាប់ដាក់ឈាម។

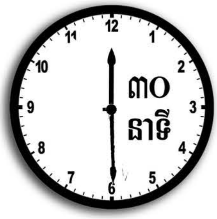

១៣-បន្តទុកបន្ទះតេស្តអោយស្ងៀមនៅកន្លែងរាបស្មើរយៈពេល ៣០នាទី។

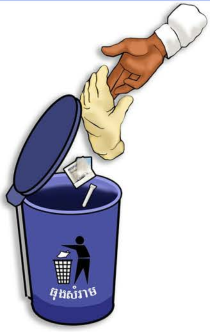

១៤-ត្រូវបោះស្រោមដៃនិងបំពង់បឺតឈាមចូលទៅក្នុងធុងសំរាមអោយបានត្រឹមត្រូវ។

INDICACIONES: Siga las instrucciones al detalle, sin saltearse ningún paso.

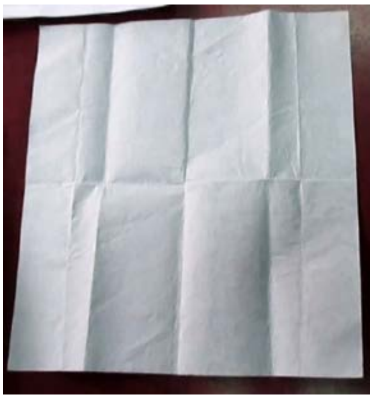

Paso 1. Coloque el campo celeste sobre la mesa con el lado blanco hacia arriba.

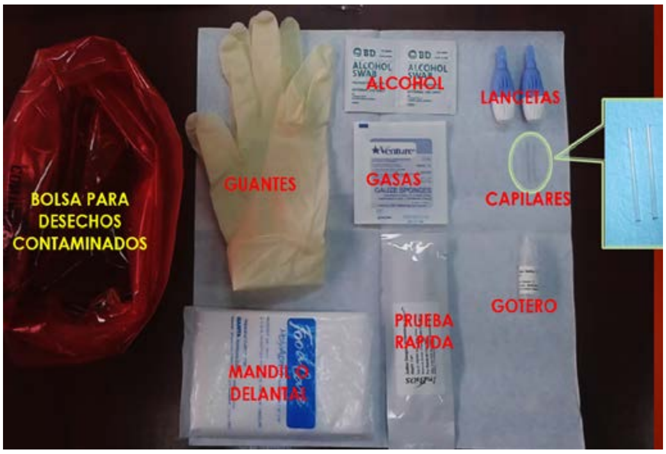

Paso 2. Coloque los materiales como en la foto y reconozca cada uno. Use la bolsa roja para todo lo que vaya botando.

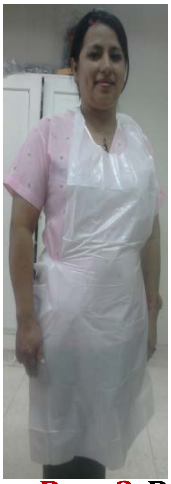
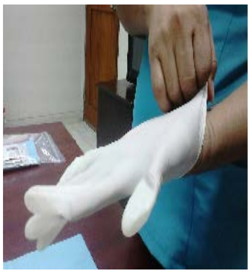

Paso 3. Póngase el mandil y los 2 guantes.

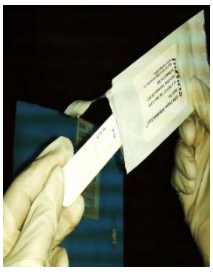
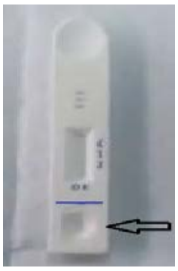

Paso 4. Saque el dispositivo de envoltura y colóquelo sobre el campo.

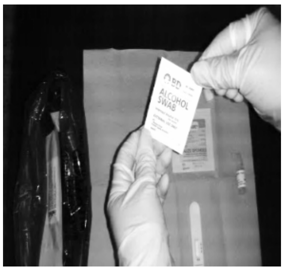

Paso 5. Abra las 3 gasas y dóblelas en 4. Después abra los 2 sobres de alcohol.

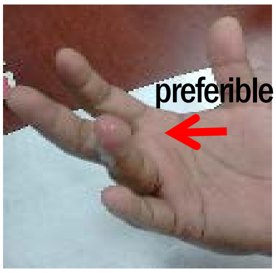

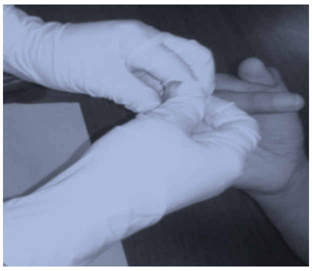

Paso 6. Ubique el dedo a picar (preferible el cuarto dedo "donde va el aro de matrimonio") y límpielo usando los 2 alcoholes.

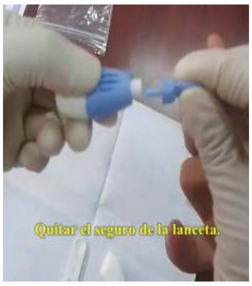

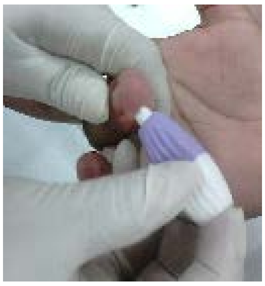

Paso 7. Gire la tapa de lanceta y jale y bote tapa. Ponga la lanceta en el costado de la yema del dedo y empuje hacia el dedo hasta oír clic, luego bote la lanceta en bolsa roja.

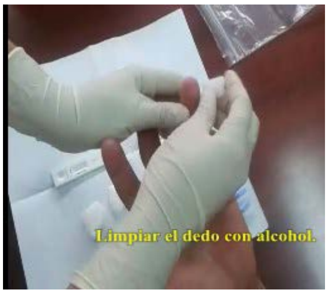

Paso 8. Limpie la primera gota de sangre con la gasa. Después masajear dedo de abajo hacia arriba para obtener otra gota.

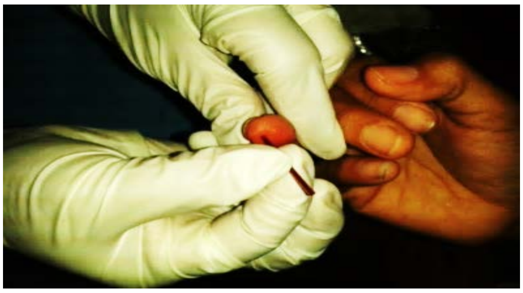

Paso 9. Colocar el tubo capilar en la gota de sangre hasta llenarlo bien. Si es necesario volver a masajear de abajo hacia arriba para obtener otra gota y colocar de nuevo el capilar. Presionar dedo picado con 1-2 gazas.

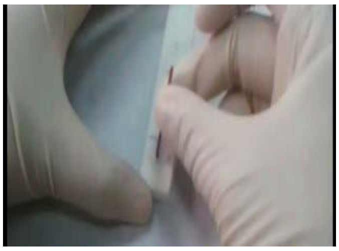

Paso 10. Poner toda la sangre del capilar en el círculo de abajo dando golpecitos con el capilar en el fondo del círculo.

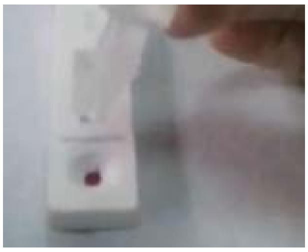

Paso 11. Poner 2 gotas del gotero en el mismo círculo.

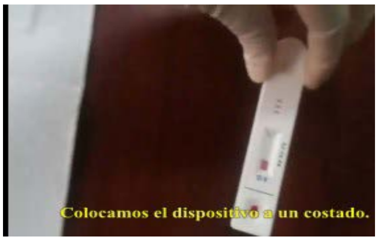

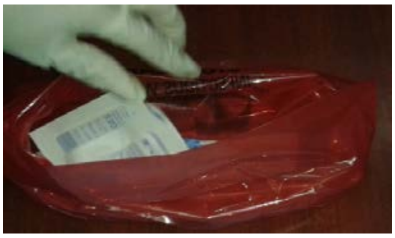

Paso 12. Ponga el dispositivo a un lado del campo. Elimine el campo, mandil, guantes y los demás materiales en la bolsa de desechos contaminados y asegúrela (Amarre). Luego observe el resultado en el dispositivo a los 30 minutos.

RESULTADOS

Raya en la letra C

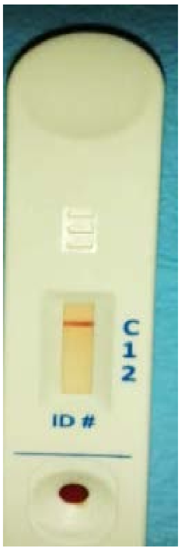

Indica que se hizo bien la prueba

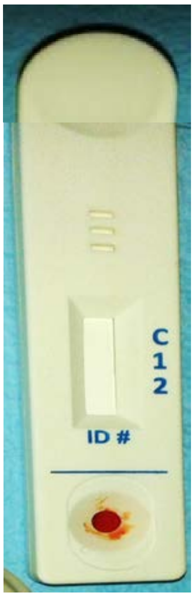

Dibuje lo que observa en el dispositivo.

INDICACIONES: Siga las instrucciones al detalle, sin saltearse ningún paso.

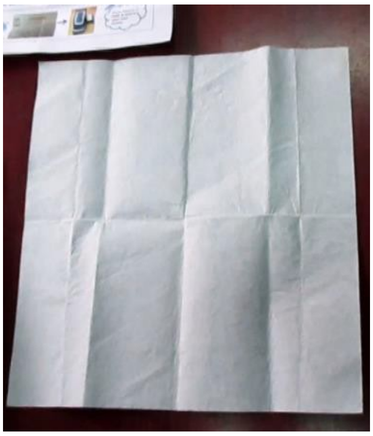

Paso 1. Coloque campo celeste sobre la mesa con el lado blanco hacia arriba.

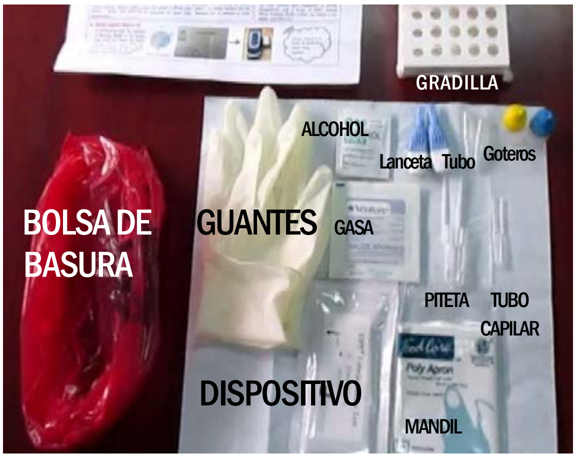

Paso 2. Coloque los materiales como en la foto y reconozca cada uno, use la bolsa roja para todo lo que vaya botando.

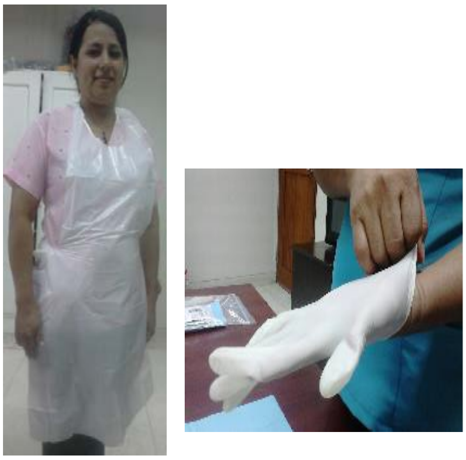

Paso 3. Póngase el mandil y los 2 guantes

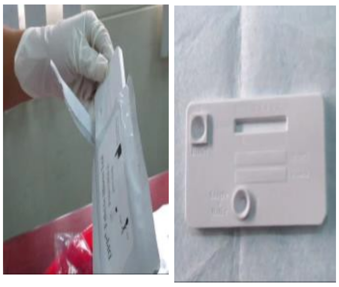

Paso 4. Abra el dispositivo. Colóquelo sobre el campo.

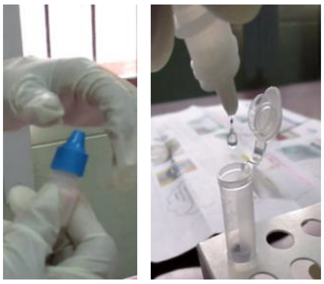

Paso 5. Agarre el gotero azul y ponga 2 gotas en el tubito y colóquelo en la gradilla.

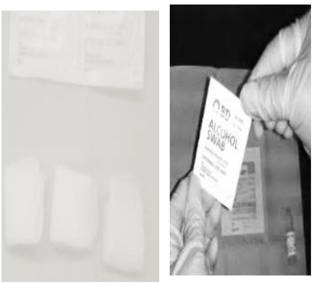

Paso 6. Abra las 3 gasas y dóblelas en 4. Después abra los 2 sobres de alcohol.

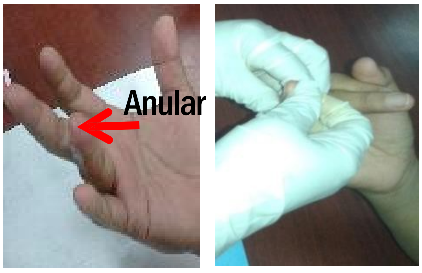

Paso 7. Ubique el dedo a picar (preferible el cuarto dedo "donde va el aro de matrimonio") y límpielo usando los 2 alcoholes.

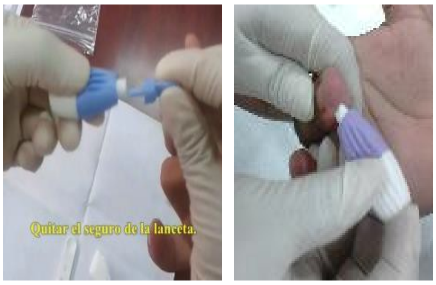

Paso 8. Gire la tapa de lanceta, jale y bote tapa. Ponga la lanceta en el costado de la yema del dedo y empuje hacia el dedo hasta oír clic, luego bote la lanceta en bolsa roja.

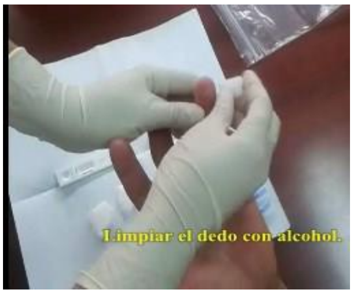

Paso 9. Limpie la primera gota de sangre con la gasa. Después masajear dedo de abajo hacia arriba para obtener otra gota.

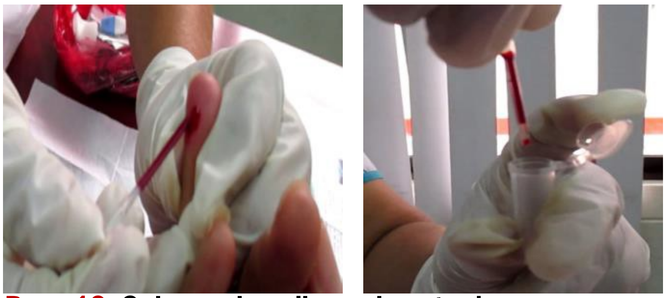

Paso 10. Colocar el capilar en la gota de sangre hasta llenarlo bien. Luego poner toda la sangre en el tubito y mézclelo bien con las gotas que se agregó anteriormente en el tubito.

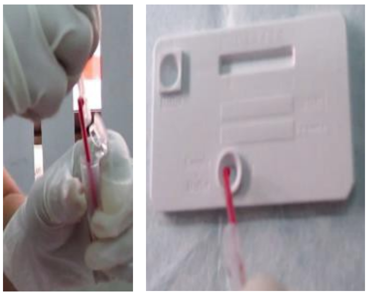

Paso 11. Agarre la pipeta y llénela con la sangre del tubito y póngalo en el círculo de abajo (Nº 1) del dispositivo y esperar 10 minutos.

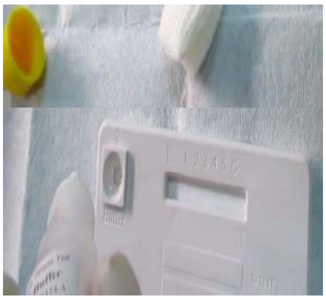

Paso 12. Poner 4 gotas del gotero amarillo en el círculo de arriba (Nº 2).

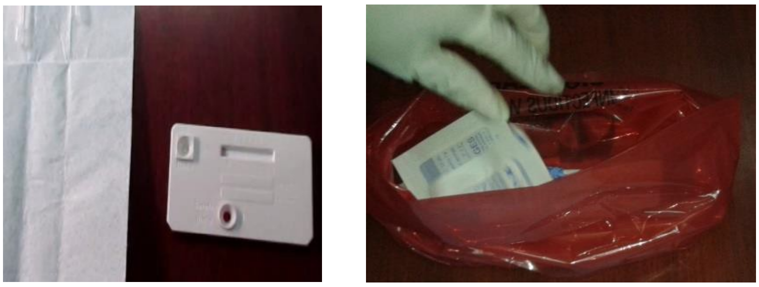

Paso 13. Ponga el dispositivo a un lado del campo. Elimine el campo, mandil, guantes y los demás materiales y amarre la bolsa. Luego observe el resultado en el dispositivo a los 20 minutos.

RESULTADOS

Raya en la letra C

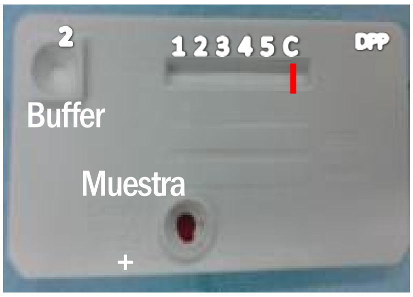

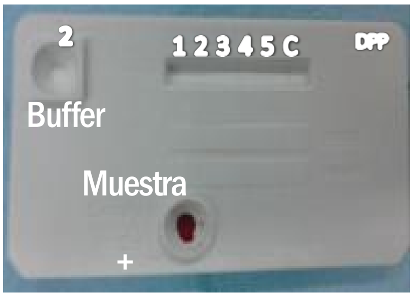

Indica que se hizo bien la prueba

Dibuje lo que observa en el dispositivo.

INDICACIONES: Prueba Rápida de Diagnostico Para Dengue NS1. Sígalo Paso a Paso.

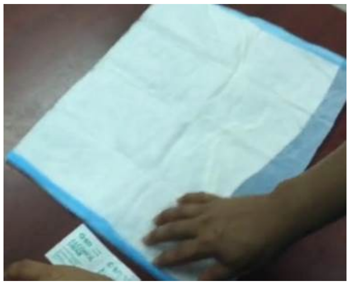

Paso 1. Coloque el campo celeste sobre la mesa con el lado blanco hacia arriba.

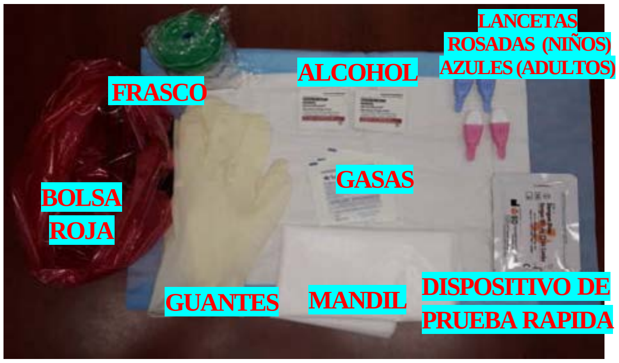

Paso 2. Coloque los materiales como en la foto y reconozca cada uno. Use la bolsa roja para todo lo que vaya botando.

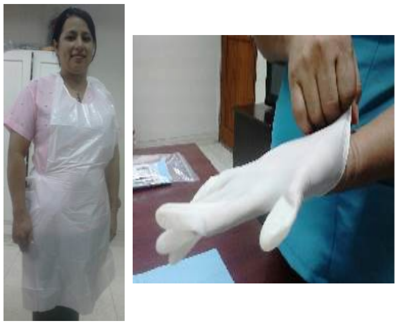

Paso 3. Póngase el mandil y los 2 guantes.

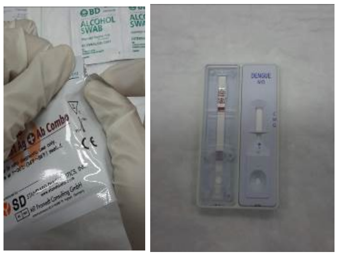

Paso 4. Abra el dispositivo. Colóquelo sobre el campo.

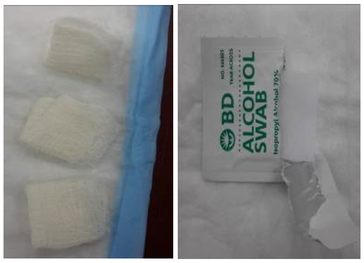

Paso 5. Abra las 3 gasas y dóblelas en 4. Después abra los 2 sobres de alcohol.

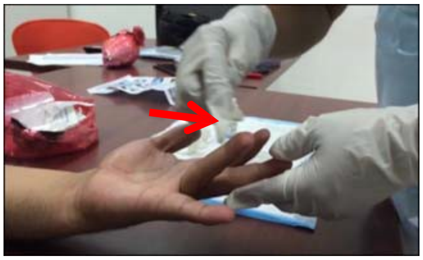

Paso 6. Ubique el dedo a picar (preferible el cuarto dedo donde va el aro de matrimonio) y límpielo usando los 2 alcoholes.

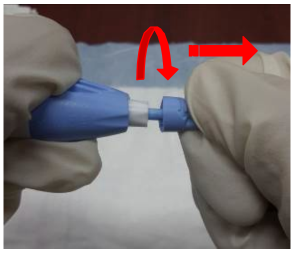

Paso 7. Elija la lanceta azul si es adulto y rosado si es niño. Vienen 2 del mismo color por si uno falla. Gire la tapa de la lanceta y jale.

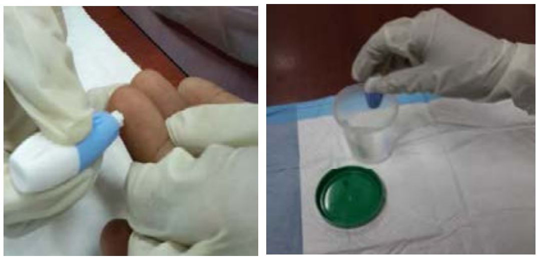

Paso 8. Ponga la lanceta en el costado de la yema del dedo y empuje hacia el dedo hasta oír clic. Luego bote la lanceta en el frasco.

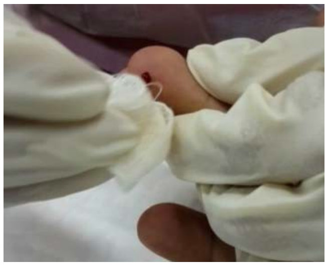

Paso 9. Limpie la primera gota de sangre con la gasa.

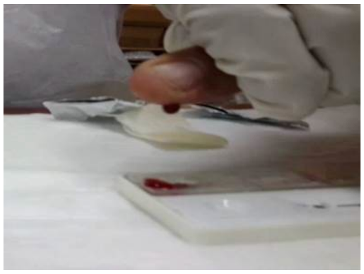

Paso 10. Dando leves masajes en el dedo, poner 2 gotas de sangre en el pocito transparente del dispositivo.

2 gotas directas del dedo aquí.

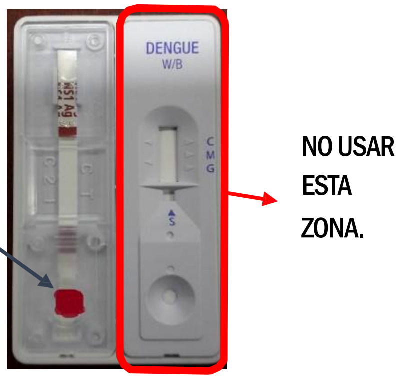

NO USAR ESTA ZONA.

Paso 11. Aquí hay una foto señalándo dónde colocar las gotas.

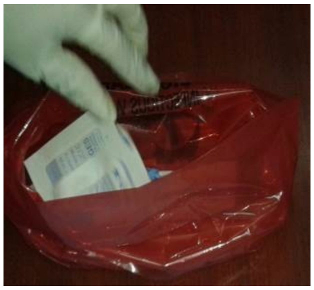

Paso 12. Elimine el campo, mandil, guantes y los demás materiales y amarre la bolsa. Luego observe el resultado en el dispositivo a los 20 minutos.

RESULTADOS

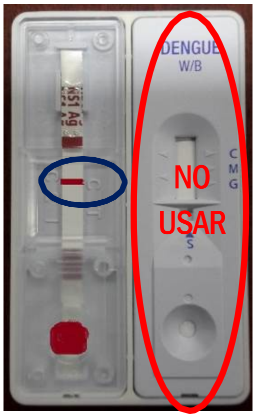

Si observa una línea roja en la letra "C" significa que la prueba fue realizada correctamente y que necesita confirmarlo con otra prueba en laboratorio.

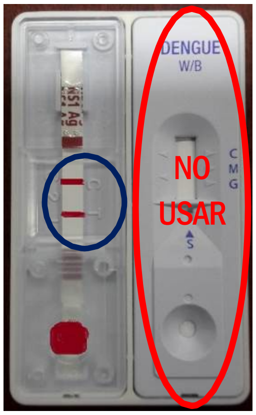

Si observa línea roja en la letra "C" y otra en la letra "T", el resultado es positivo. Hay evidencia de Dengue.

"Cualquiera que sea su resultado, por favor llamar a la persona que lo está visitando e infórmele del resultado"

ENCARGADOS

NOMBRE	CELULAR	RPM
[redacted]	[redacted]	[redacted]
[redacted]	[redacted]	[redacted]
[redacted]	[redacted]	[redacted]
[redacted]	[redacted]	[redacted]

Jhonny
D:20170221094343-05'00'2/21/2017 9:43:43 AM

Accepted set by Jhonny
