# Supplementary material for: Potential for community based surveillance of febrile diseases: Feasibility of self-administered rapid diagnostic tests in Iquitos, Peru and Phnom Penh, Cambodia
Source: PLoS Negl Trop Dis. 2021 Apr 26;15(4):e0009307. doi: 10.1371/journal.pntd.0009307 (PMC8101991; doi:10.1371/journal.pntd.0009307)
Supplement: S2 Text — (DOCX) [file pntd.0009307.s002.docx]

**S2. Video instructions provided to community members on DVD to perform self-testing for two-line (**<https://youtu.be/5RQtc9KNA5A>**), five-line (**<https://youtu.be/adv-yQaI_iA>**) used for observational competency studies and the SDBioline Dengue Duo** <https://youtu.be/arOSOaeulIw>

Note: If excepted we will attempt to provide the video to be loaded on the PLOS server. With COVID our teams in Iquitos have been on lock down and unable to access their computers.
