# Supplementary material for: Potential for community based surveillance of febrile diseases: Feasibility of self-administered rapid diagnostic tests in Iquitos, Peru and Phnom Penh, Cambodia
Source: PLoS Negl Trop Dis. 2021 Apr 26;15(4):e0009307. doi: 10.1371/journal.pntd.0009307 (PMC8101991; doi:10.1371/journal.pntd.0009307)
Supplement: S3 Text — (DOCX) [file pntd.0009307.s003.docx]

Table S3. Observational data collection sheet used by study observers

|  | The participant… | No/ poorly | Medium | Yes/ good | Needed help | Comments |
| --- | --- | --- | --- | --- | --- | --- |
| 1 | Recognizes and differentiates materials | 1 | 2 | 3 | No … 0  Yes … 1 |  |
| 2 | Was able to locate a puncture site | 1 | 2 | 3 | No … 0  Yes … 1 |  |
| 3 | Properly cleaned the puncture site with alcohol before using the lancet. | 1 | 2 | 3 | No … 0  Yes … 1 |  |
| 4 | Used the lancet correctly | 1 | 2 | 3 | No … 0  Yes … 1 |  |
| 5 | Placed the capillary correctly on finger | 1 | 2 | 3 | No … 0  Yes … 1 |  |
| 6 | Collected enough blood for the test | 1 | 2 | 3 | No … 0  Yes … 1 |  |
| 7 | Located the “well” for blood on device | 1 | 2 | 3 | No … 0  Yes … 1 |  |
| 8 | Transferred all the blood into the well | 1 | 2 | 3 | No … 0  Yes … 1 |  |
| 9 | Correctly added buffer to the well | 1 | 2 | 3 | No … 0  Yes … 1 |  |
| 10 | Waited >30 mins to read results | 1 | 2 | 3 | No … 0  Yes … 1 |  |
| 11 | Located the location on device to read results | 1 | 2 | 3 | No … 0  Yes … 1 |  |
| 12 | Correctly marked the results card | 1 | 2 | 3 | No … 0  Yes … 1 |  |
| 13 | Referred to written instructions while using device | 1 | 2 | 3 | No … 0  Yes … 1 |  |
| 14 | Liked the concept of the device | 1 | 2 | 3 | No … 0  Yes … 1 |  |
| 15 | Applied the device without needing motivation | 1 | 2 | 3 | No … 0  Yes … 1 |  |
| 16 | Applied device without being stressed or nervous | 1 | 2 | 3 | No … 0  Yes … 1 |  |
| 17 | Was satisfied with the overall experience | 1 | 2 | 3 | No … 0  Yes … 1 |  |
